# The first-in-class pro-apoptotic peptide PEP-010 is effective in monotherapy and in combination with paclitaxel on resistant ovarian adenocarcinoma cell models

**DOI:** 10.3389/fphar.2024.1444973

**Published:** 2024-08-07

**Authors:** Aline Lacroix, Rayan Farhat, Aude Robert, Catherine Brenner, Joëlle Wiels, Diego Germini

**Affiliations:** ^1^ PEP-Therapy, Paris, France; ^2^ UMR1279 INSERM, Université Paris-Saclay, Villejuif, France; ^3^ CNRS UMR9018, Metabolic and Systemic Aspects of Oncogenesis for New Therapeutic Approaches, Université Paris-Saclay, Villejuif, France

**Keywords:** apoptosis, peptide, monotherapy, combination therapy, ovarian adenocarcinoma

## Abstract

Ovarian adenocarcinoma is the gynecological malignancy with the worst prognosis and the highest mortality rate. In the first stages of treatment, chemotherapy results effective, but its prolonged use and high doses lead to the appearance of resistance to treatments and relapse in most patients, representing a major challenge for clinicians. We developed PEP-010, a cell penetrating proapoptotic peptide disrupting the protein-protein interaction between caspase-9 and protein phosphatase 2A, thereby leading to the recovery of their activity in the apoptotic pathway. MTT assay or Annexin-V/Propidium Iodide staining and flow cytometry analysis were used to assess sensitivity to chemotherapies and apoptosis after treatment with PEP-010 in monotherapy or in combination with paclitaxel in ovarian carcinoma cell lines. DNA damage was assessed by immunofluorescence using γH2AX marker. We show here that PEP-010 effectively induces cell death in monotherapy on in up to 55% of cells from ovarian adenocarcinoma cell models resistant to different chemotherapies. Moreover, when used in combination with paclitaxel, one of the therapeutic options for recurrent ovarian carcinoma, PEP-010 showed a beneficial effect leading to the reduction of the IC_50_ of paclitaxel of 2.2 times and to apoptosis in 87% of cells. The described results suggest the potential therapeutic interest for PEP-010 and lead to the choice of ovarian adenocarcinoma as one of the major indications of the ongoing clinical trial.

## Introduction

Ovarian adenocarcinoma (OA) has the worst prognosis and the highest mortality rate among gynecological malignancies, with less than 50% average 5-year survival (Phung et al., 2023). OA treatment relies on the tumor stage, including surgical removal and platin- or taxane-based chemotherapies. Although chemotherapy is initially effective, prolonged use and high doses lead to the appearance of resistance and relapse in ∼70% of patients (Alatise et al., 2022). Drug resistance is a multi-factorial mechanism. However, the most widely accepted molecular mechanism is the dysregulation of both influx and efflux pumps (multi-drug resistance (MDR) pumps), regulating the transport of compounds in cancer cells (Ortiz et al., 2022).

Strategies to overcome this issue are required. A promising strategy for novel anticancer treatment can be to specifically act on proteins of the apoptotic pathway with the aim of re-establishing the normal ability for a cell to die (Peng et al., 2022). FDA approval of venetoclax in 2016, a pro-apoptotic small molecule inhibitor of Bcl-2, for the treatment of chronic lymphocytic leukemia or acute myeloid leukemia, paved the way for targeting apoptosis in cancer therapy (Carneiro and El-Deiry, 2020). Among the possible therapeutic strategies, peptides are gaining strong interest. Indeed, they allow targeting specifically pathological protein–protein interaction, which is difficult to target with small molecules (Anand et al., 2023). Peptide-based drugs have been FDA-approved both for cancer (as antibody-drug conjugates or labeled peptides for diagnostic use) and other diseases (e.g., diabetes or cardiovascular diseases, among others) (de la Torre and Albericio, 2020; Wang et al., 2022). Other peptide drugs with different downstream targets and mechanisms of action are being investigated in clinical/preclinical trials (Urandur and Sullivan, 2023; Vadevoo et al., 2023). Furthermore, the use of cell-penetrating peptides as carriers for various types of therapeutic molecules is also being explored to overcome the drawbacks of standard chemotherapy (Xie et al., 2020; Matijass and Neundorf, 2021).

We developed PEP-010, a pro-apoptotic, bifunctional peptide with cell-penetrating and interfering peptide capacity. These molecules efficiently penetrate cells and specifically block intracellular protein–protein interactions, leading to the inhibition of key pathological mechanisms without altering physiological mechanisms and re-establishing cellular pathways. Upon cell penetration, PEP-010 disrupts the interaction between caspase-9 and protein phosphatase 2A (PP2A), two key proteins involved in apoptosis, a physiological process frequently altered in cancer.

Previous studies showed a rapid entry of PEP-010 followed by apoptosis induction in several cancer cell lines of different tumor origins (Arrouss et al., 2013; Farhat et al., 2023). In this study, we demonstrated the pharmacological potential of PEP-010 for OA treatment in monotherapy and in combination with paclitaxel (PTX), a chemotherapy drug indicated for platinum-resistant OA treatment. The results obtained in this work paved the way for the ongoing clinical trial (NCT04733027), which thus focuses on platin-resistant OA.

## Materials and methods

### Cell culture

The ovarian cell lines IGROV1, IGROV1CDDP, and IGROV1VCR were cultivated in RPMI 1640 Medium GlutaMAX with 10% FBS and 1% of pyruvate and glutamine. They were maintained at 5% CO_2_ and 37°C in a humidified atmosphere. All reagents are from Gibco^®^ (Thermo Fisher Scientific, Carlsbad, CA, United States).

### Peptides

PEP-010 sequence is VKKKKIKAEIKIYVETLDDIFEQWAHSEDL, where VKKKKIKAEIKI is the cell-penetrating part of the peptide and YVETLDDIFEQWAHSEDL is the interfering peptide part. PEP-010 was produced by PolyPeptide (Strasbourg, France).

### Cell treatment

PEP-010 was first dissolved in 0.1% formic acid (10 mM) and then diluted in a cell culture medium at pH9 (to allow a full dissolution of the peptide). Experiments in similar conditions, but without the peptide, were conducted as negative controls. Staurosporine (Selleckchem, Houston, TX, United States) was used as a positive control at a concentration of 1 µM. Cells were collected or analyzed at the indicated time points. For combination conditions, the cells were treated with paclitaxel (Accord, London, United Kingdom) at the indicated concentrations for 72 h.

### MTT assay

Here, 10,000 cells were seeded on 96-well plates in 100 µL of medium. The day after, the medium was replaced with fresh medium containing the appropriate drugs at the indicated concentrations: cisplatin (Mylan, Canonsburg, PA, United States), paclitaxel (Accord, London, United Kingdom), doxorubicin (Pfizer, New York, NY, United States), vincristine [Pfizer, New York, NY, United States)], and/or PEP-010. The plates were incubated at 37° in humidified air with 5% CO_2_ for 72 h. The MTT reagent (20 µL) was added at the end of the indicated time points, and plates were incubated for 2 h at 37°C. In addition, 100 µL of lysis buffer was added to each well. Subsequently, the plates were incubated for 2 h at 37°C, and the absorbance was measured at 570 nm (Tecan, Männedorf, CH).

### Apoptosis measurement

Cell death was assessed using Annexin V-APC and propidium iodide (PI) (BioLegend, San Diego, CA, United States) staining following the manufacturer’s instructions. Results were analyzed as previously described (Debernardi et al., 2018). A total of 10,000 events were analyzed using a C6 Accuri Cytometer (Becton-Dickinson, Franklin Lakes, NJ, United States), analysis was performed using integrated software, and Annexin V positive cells (PI positive and negative) were counted as apoptotic.

### Immunofluorescence

Cells were seeded on 12 mm coverslips placed in a 24-well plate (24 h, 37°C, and 5% CO_2_). Cells were treated with PEP-010 as previously described. At the indicated time point, cells were washed with 1X PBS and fixed in 4% paraformaldehyde (Euromedex, Souffelweyersheim, FR) in 1X PBS (10 min, RT), followed by three washes in 1X PBS. Cells were then permeabilized with 2% Triton X-100 (Euromedex, Souffelweyersheim, FR) in 1X PBS (10 min, RT) and then washed in 1X PBS. Cells were saturated with 0.5% BSA (Euromedex, Souffelweyersheim, FR) in 1X PBS (40 min, RT) and then stained with an anti-γH2AX Ser139 antibody (mouse, 1:200, from BioLegend #613402). Incubation with primary antibody (2 h, RT) was followed by three washes in 1X PBS and by incubation (1 h, RT) with a secondary antibody conjugated to Alexa Fluor 588 goat anti-mouse (Thermo Fisher Scientific, Carlsbad, CA, United States), diluted 1:200. Coverslips were mounted on microscopy glass slides using a mounting medium containing DAPI (Sigma-Aldrich, Saint-Quentin-Fallavier, Cedex, France). Images were acquired using a fluorescence microscope (Zeiss Observer Z1). Images were analyzed using ImageJ software.

### Statistics

For each time point, different treatments are compared with the control analyzed at the same time point. Data are presented as the mean ± standard error of the mean (SEM). A student’s t-test (two-tail distribution) was used to compare the means of the two groups (treated vs. untreated or monotherapies between each other or vs. combination). All tests were performed using GraphPad Prism 5 software (GraphPad Prism, Boston, MA, United States).

## Results

To evaluate the efficacy of PEP-010 for OA treatment, we used three OA cell lines, the parental IGROV1 established from a stage III ovarian primary tumor and two *in vitro* derived drug-resistant cell lines: IGROV1CDDP, resistant to cisplatin, and IGROV1VCR, resistant to vincristine. The latter expresses MDR pumps (Supplementary Figure 1A, B).

**FIGURE 1:**
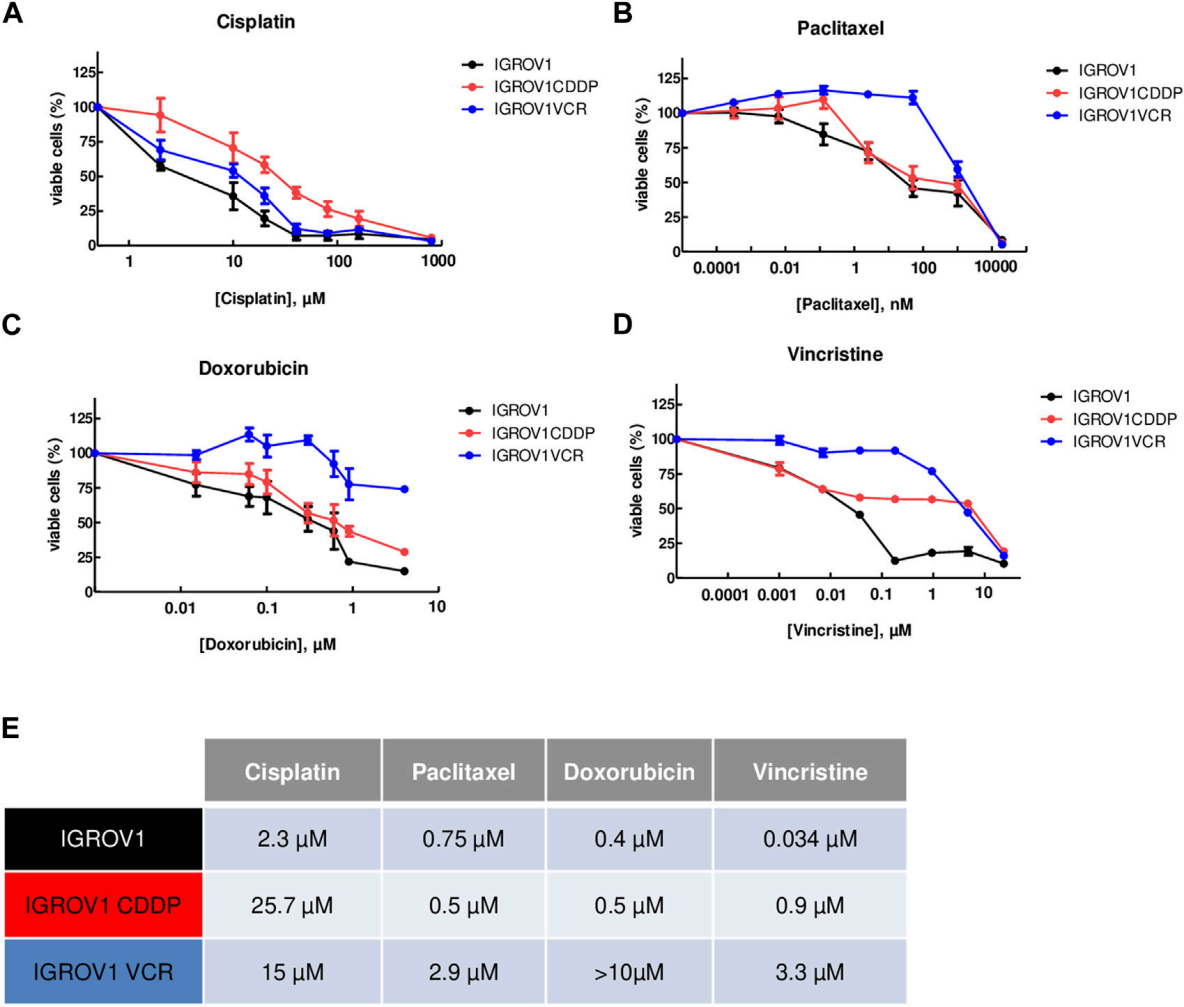
OA cell lines used are resistant to different chemotherapies. **(A–D)** Calculation of IC_50_ values using the MTT assay upon treatment with cisplatin **(A)**, paclitaxel **(B)**, doxorubicin **(C)**, or vincristine **(D)** for 72 h. IGROV1 survival percentages are represented by black dots connected by a black line, IGROV1CDDP is represented in red, and IGROV1VCR is represented in blue. All experiments have been performed on three to six independent biological replicates. For each point, the mean ± SEM is represented. **(E)** IC_50_ values obtained in the different models and with the indicated conditions. The table shows the values obtained in IGROV1, IGROV1CDDP, and IGROV1VCR with cisplatin, paclitaxel, doxorubicin, or vincristine.

The resistance of IGROV1CDDP and IGROV1VCR to widely used chemotherapies, cisplatin (CDDP), paclitaxel (PTX), doxorubicin (DOX), and vincristine (VCR), was assessed by IC_50_ determination using the MTT test and compared to the parental line IGROV1. As expected, IGROV1CDDP were highly resistant to CDDP (IC_50_ = 25.7 µM ± 4.6; fold change (FC) vs*.* IC_50_ IGROV1 = 11.1, Figure 1A), while IGROV1VCR showed resistance to all tested chemotherapies (IC_50_ CDDP = 15 µM ± 4, FC = 6.5; IC_50_ PTX = 2.9 µM ± 0.6, FC = 3.9; IC_50_ DOX>10µM; and IC_50_ VCR = 3.3 µM ± 1.5, FC = 97, Figures 1A–D). IC_50_ values are reported in Figure 1E.

**FIGURE 2:**
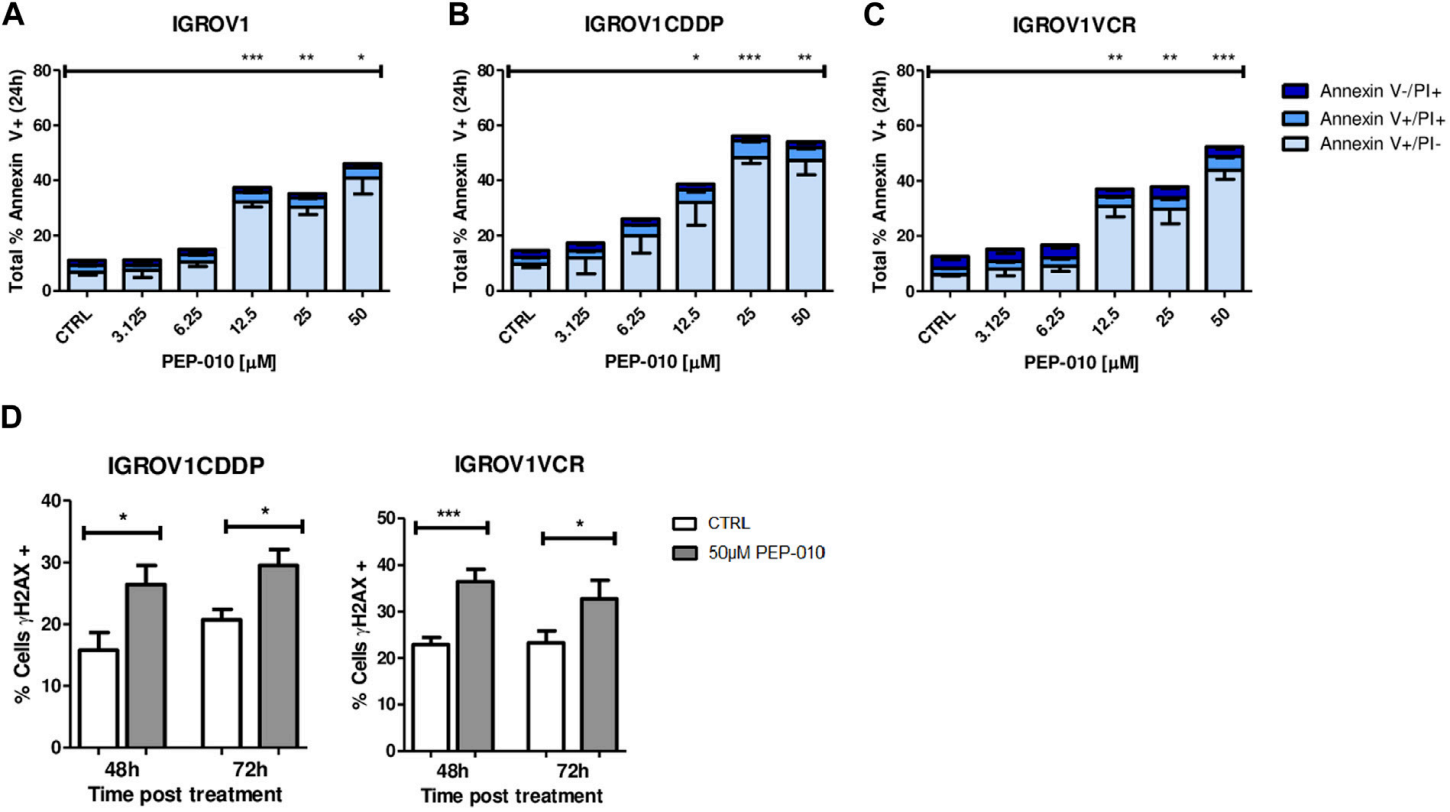
PEP-010 induces apoptosis in OA cells resistant or not to chemotherapies. **(A–C)** Cells were treated with PEP-010 at the indicated doses for 24 h. Results of treated cells were always compared to those of the untreated control. Analysis of cell death was performed by Annexin V/PI staining and FACS analysis. The results are represented as the total percentage of cells expressing Annexin V. All experiments have been performed in three independent biological replicates. Early apoptosis (Annexin V+/PI-) is represented in light blue; late apoptosis (Annexin V+/PI+) is represented in blue; and necrosis (Annexin V-/PI+) is represented in dark blue. **(D)** PEP-010 induces DNA damage, as shown by γH2aX expression. Fixed cells were immunostained using an antibody specific for γH2aX as an early marker of DNA damage. The percentage of cells expressing γH2aX was calculated over the total number of cells. Three biological and nine technical replicates and at least 300 cells were analyzed for each biological replicate. Graphs represent the means ± SEM. *, <0.05; **, 0.001 < *p* < 0.05; and ***, *p* < 0.001.

Then, we assessed whether PEP-010 could induce apoptosis in these cell models by Annexin V/PI staining and FACS analysis. Starting from 12.5 µM, total Annexin V levels were significantly higher in all three models at 24 h post-treatment than in the untreated control (total % of Annexin V positive cells: 37.4 ± 1.9 vs*.* 11 ± 1.3 for IGROV1, *p* = 0.0004, Figure 2A; 38.7 ± 8.5 vs*.* 14.6 ± 1.2 for IGROV1CDDP, *p* = 0.046; Figure 2B; and 37 ± 4 vs*.* 12.6 ± 0.9 for IGROV1VCR, *p* = 0; 007; Figure 2C). Thus, PEP-010 shows an antitumor effect on these cells independently of their drug resistance status and MDR pump expression.

As previously mentioned, the appearance of resistance is a major cause of failure of OA treatment; therefore, we focused our attention on resistant models. As a further confirmation of the PEP-010 apoptosis induction, we assessed the presence of DNA damage (DD) by γH2AX staining followed by immunofluorescence analysis. γH2AX expression was increased upon treatment with PEP-010 as compared to untreated controls in all tested models (% of γH2AX positive cells 48 h post-treatment = 26.5 ± 3.1 vs*.* 15.8 ± 2.8 for IGROV1CDDP and 29.5 ± 2.6 vs*.* 20.7 ± 1.7 for IGROV1VCR, Figure 2D). Taken together, our data establish that PEP-010 effectively induces apoptotic cell death in different OA models independently of potential resistance mechanism of the cell lines.

**FIGURE 3:**
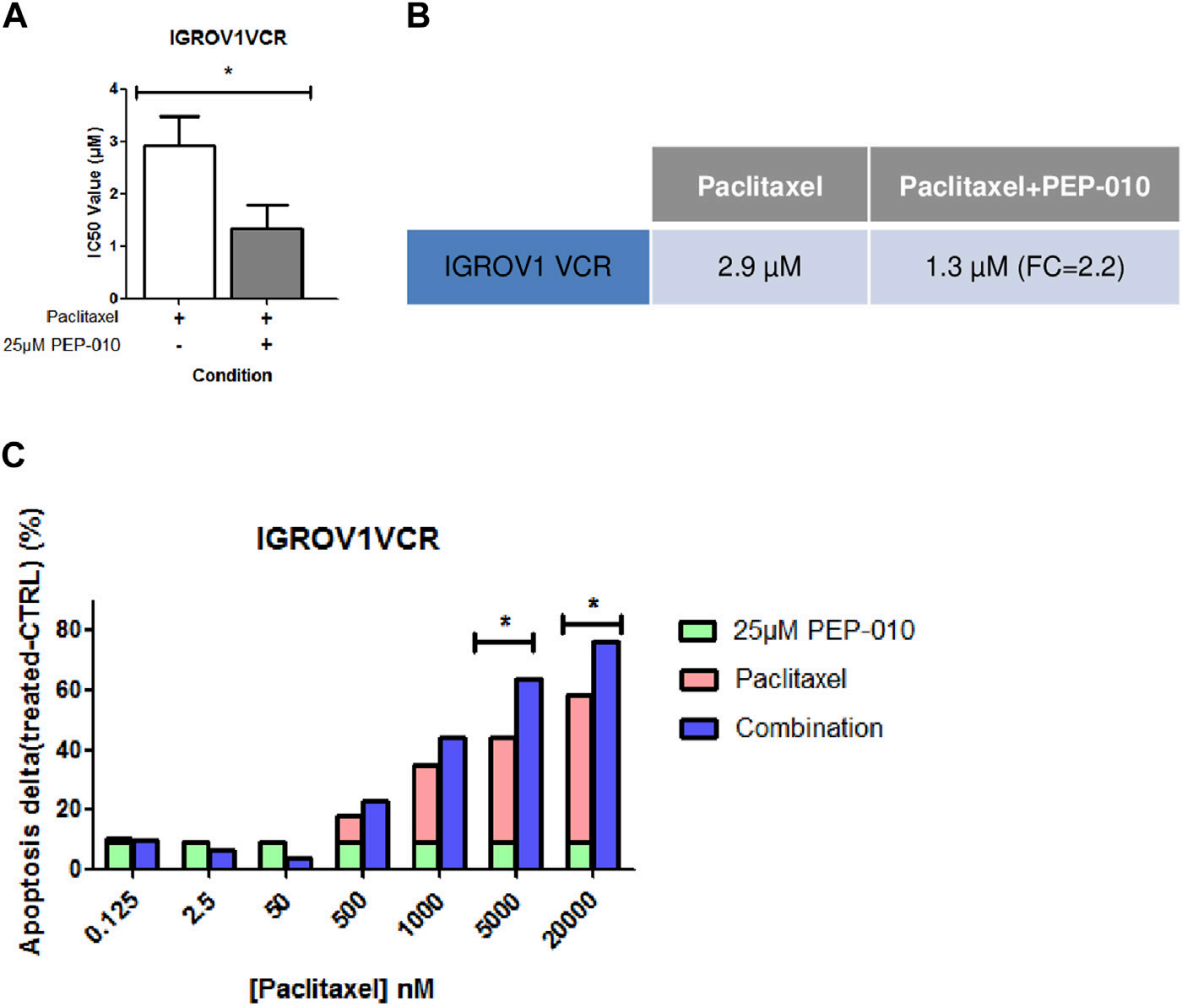
PEP-010 combination with paclitaxel shows a beneficial effect on OA cell lines resistant to chemotherapies. **(A, B)** IC_50_ values obtained by calculation using the MTT assay upon treatment of IGROV1VCR with the indicated drugs for 72 h. The IC_50_ value decreases when PEP-010 is added to paclitaxel. Four independent biological replicates have been analyzed. **(C)** The combination effect was also analyzed by Annexin V/PI staining in IGROV1VCR treated with increasing doses of paclitaxel in monotherapy or with the addition of PEP-010 (25 µM) for 72 h. Three independent biological replicates have been analyzed. *, <0.05; **, 0.001 < *p* < 0.05; and ***, *p* < 0.001.

A combination of drugs targeting various molecular pathways could decrease the therapeutic dose of each chemotherapeutic agent and thereby delay or avoid resistance acquisition (Kuosmanen et al., 2021). We thus assessed, by MTT assay, whether PEP-010 could improve PTX efficacy (i.e*.,* the current chemotherapy indicated for platinum-resistant OA treatment) by monitoring the simultaneous use of PEP-010 with PTX on IGROV1VCR. Cells were treated for 72 h with increasing doses of PTX alone or in combination with PEP-010 used at a single concentration (25 µM). We observed that the treatment with PEP-010 was advantageous as it significantly decreased the IC_50_ value of PTX (PTX alone = 2.9 µM ± 0.6 vs. PTX + PEP-010 = 1.3 µM ± 0.5, FC = 2.2 (Figure 3A,B). These data were further confirmed by Annexin V/PI staining, where an additive effect and a dose-effect correlation on apoptosis were visible starting from 500 nM PTX (combination effect = +4.9% vs. monotherapy with PTX 500 nM, +9.4% at 1 µM, +19.5% at 5 µM, and +18% at 20 µM) (Figure 3C). The combination index is < 1, confirming a synergic effect of the two drugs. The beneficial combination of paclitaxel with pro-apoptotic drugs is consistent with previous observations in other cancer models. This is notably the case of navitoclax (an inhibitor of BCL-2, BCL-xL, and BCL-W), which had a high synergy rate with paclitaxel in some subtypes of breast cancer (Jaaks et al., 2022).

## Discussion

Ovarian cancer is the eighth most common cancer in women worldwide and remains the leading cause of death among gynecological cancers. The standard of care is a debulking strategy followed by platin- or taxane-based chemotherapy (Arora et al., 2024). Unfortunately, a majority of patients relapse, and among them, two subpopulations could be distinguished: platinum-sensitive patients, who could receive platinum-based chemotherapies again, often followed by PARP inhibitor treatments, or platinum-resistant patients, for whom cancer recurs within 6 months after the end of chemotherapy, with a median survival rate of 9–12 months. Resistance to cisplatin could pre-exist (approximately 20% of patients do not respond to cisplatin) or, in 70%–80% of the cases, be acquired during treatment (Baert et al., 2021; Awada et al., 2022; Blagden and Nicum, 2021). These patients are therefore in great need of new therapeutic tools, but most of the recently developed approaches have the same strategy as the treatments already approved (immunotherapies, VEGF inhibitors, PARP inhibitors, and folate alpha inhibitors) (Satora et al., 2024). Therefore, the development of novel therapies with novel targets is required.

Dysregulation of influx/efflux pump functionality is one of the most widely accepted platinum resistance mechanisms (Ortiz et al., 2022). At the molecular level, the ability of cancer cells to escape apoptosis is one of the most common ways to resist treatments (Neophytou et al., 2021). For this reason, re-teaching the cell to die is a promising strategy to overcome the resistance issue.

A novel therapeutic opportunity is represented by peptides for their specific targeting of protein–protein interactions. In this regard, we show in this study that PEP-010, a pro-apoptotic peptide targeting and disrupting the interaction between PP2A and caspase-9, efficiently induces apoptosis in OA cell lines resistant to different chemotherapeutic drugs *in vitro*, independently of MDR pump expression. PEP-010 efficacy relies on the restoration of the physiological roles of PP2A and caspase-9 when they are released. On one side, PP2A can regulate and inactivate a large amount of apoptosis/pro-survival-related downstream targets (e.g., Bcl-2 family members and Akt) (Farrel et al., 2014), and on the other side, caspase-9, once activated, can trigger the caspase cascade leading to cell death.

A strategy to reduce or delay the onset of resistance and minimize the toxicity of chemotherapies is to combine them with other drugs. To this aim, we combined PEP-010 with PTX and demonstrated a beneficial effect of this combination, leading to a reduction in the IC_50_ value of PTX and an increased apoptotic effect compared to monotherapy, thereby revealing a potential therapeutic interest. These results obtained on cell lines could be strengthened in the future by performing *in vivo* studies using PEP-010 in combination with paclitaxel or with other therapies (e.g., PARP inhibitors, which would allow targeting the apoptosis pathway at different levels).

However, it must be mentioned that PEP-010 safety has been evaluated in monotherapy and in combination with chemotherapy during a Phase 1a study, where patients with solid tumors, including OA, were enrolled.

Finally, the data described in this paper served as the basis for the choice of the therapeutic indication for Phase 1b of the PEP-010 ongoing clinical trial (NCT04733027), which thus focuses on platin-resistant OA and pancreatic adenocarcinoma (Rusquec et al., 2023).

## Data Availability

The raw data supporting the conclusions of this article will be made available by the authors, without undue reservation.
