# Supplementary figures and images for: The first-in-class pro-apoptotic peptide PEP-010 is effective in monotherapy and in combination with paclitaxel on resistant ovarian adenocarcinoma cell models
Source: Front Pharmacol. 2024 Aug 7;15:1444973. doi: 10.3389/fphar.2024.1444973 (PMC11335512; doi:10.3389/fphar.2024.1444973)

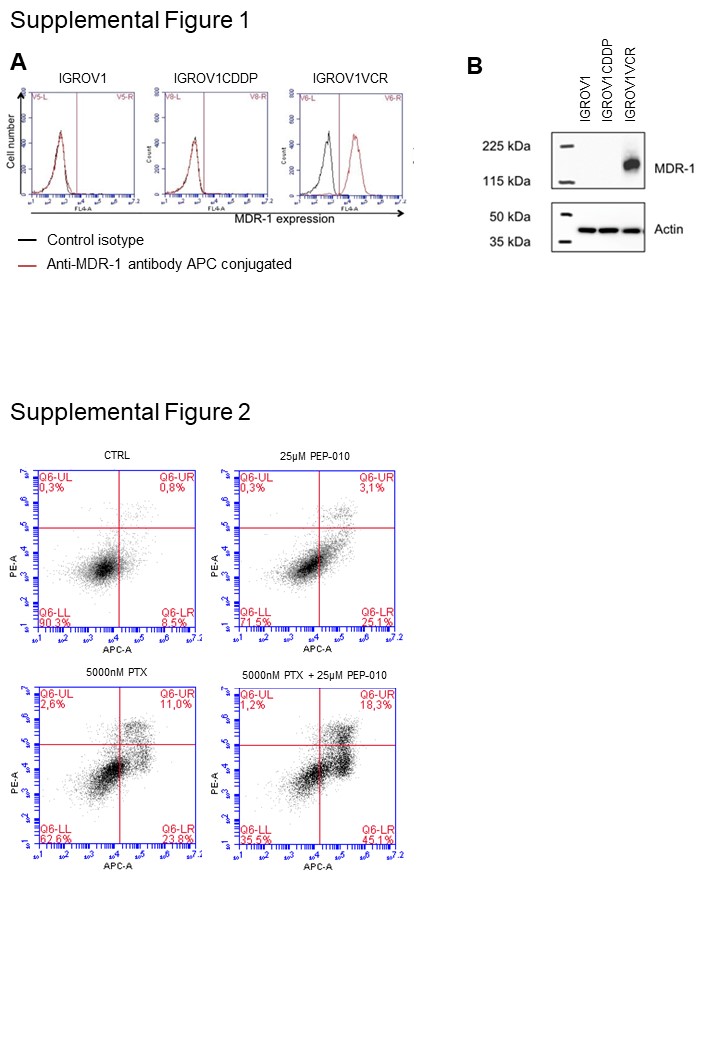

Supplement: Supplementary file 1 [file Image1.JPEG]
