# Supplementary material for: The first-in-class pro-apoptotic peptide PEP-010 is effective in monotherapy and in combination with paclitaxel on resistant ovarian adenocarcinoma cell models
Source: Front Pharmacol. 2024 Aug 7;15:1444973. doi: 10.3389/fphar.2024.1444973 (PMC11335512; doi:10.3389/fphar.2024.1444973)
Supplement: Supplementary file 2 [file DataSheet1.docx]

**Supplemental Material**

**Supplemental Figures**

**Supplemental Figure 1. IGROV1VCR expresses MDR pumps.** Analysis of MDR-1 expression on IGROV1 parental and derived cell lines. (**A**) Surface analysis of MDR-1 expression by flow cytometry. Plots represent results obtained on cells labelled with isotype control (represented in black) or with an antibody against MDR-1 (represented in red). (**B**) Western blot analysis of MDR-1 expression (170 kDa) on whole cell extracts. Actin (42 kDa) was used as a loading control. Presented results are from one representative experiment.

**Supplemental Figure 2.** Representative dot-plot of cells treated with the indicated conditions and stained with Annexin V/PI for flow cytometry analysis.

**Supplemental Methods**

**Immunofluorescence with FACS.** Cells were fixed and permeabilized in 70% ethanol in 1X PBS overnight. The day after, cells were centrifuged 5min at 800g, then cells were washed 1 time with 1X PBS and 1 time with BSA 0.5% to remove ethanol residues. Cells were incubated with the primary antibody anti-MDR1 (Rabbit 1:300, 150 kDa, ThermoFisher Scientific, Carlsbad, CA, USA) diluted in 0.5% BSA - 0.5% Tween20 for 30min RT, and then they were washed 1 time with 0.5% BSA and centrifuge for 5min at 800g followed by incubated with the secondary antibody (Alexa Fluor 647 Goat anti-rabbit IgG used 1:200, ThermoFisher Scientific, Carlsbad, CA, USA) diluted in 0.5% BSA – 0.5% Tween20 for 30min RT in the dark. Cells were washed 1 time with 0.5% BSA and centrifuge for 5min at 800g. The pellet was mixed in 100µl 1X PBS. 10.000 events were analyzed by C6 Accuri cytometer (Becton-Dickinson, Franklin Lakes, NJ, USA).

**Western blot.** Total protein lysates were prepared from desired cells using NETN buffer (NaCl 150mM, Tris pH7.5 50mM, NP40 0.5%, Phosphatase inhibitor (Sigma-Aldrich, Saint-Quentin-Fallavier Cedex

France) and Protease inhibitor cocktail (Sigma-Aldrich, Saint-Quentin-Fallavier Cedex

France), then sonicated 10 sec, 30% of intensity to fully break the cells. Lysates were then incubated on ice for 30min and centrifuged at 4°C maximum speed for 10min.

The proteins were quantified using the Pierce BCA protein assay kit (ThermoFisher Scientific, Carlsbad, CA, USA) with absorbance measured at 562 nm with a NanoDrop 2000c (ThermoFisher Scientific, Carlsbad, CA, USA). The protein fractions thus obtained were mixed with the NuPAGE LDS Sample Buffer 4x (ThermoFisher Scientific, Carlsbad, CA, USA), denatured (95 °C, 5 min), loaded onto a 4–12% Bis-Tris gel (Sigma-Aldrich, Saint-Quentin-Fallavier Cedex, France) and run at 80V, 30min then 120V, 1h at RT. Proteins were transferred from the gels onto Bio-RAD Nitrocellulose membranes 0,2µM transfer membrane (Bio-RAD, Hercules, CA, USA) at 90V for 2h at 4°. After transfer the membrane was blocked in 5% milk in TBS. After blocking, the membrane was incubated with primary antibodies anti-MDR1 (Rabbit 1:300, 150 kDa, ThermoFisher Scientific, Carlsbad, CA, USA) and anti-Actin (Mouse 1:1000, 43 kDa; Santa Cruz Biotechnology, Dallas, TX, USA, used as loading control) overnight, 4°C. After three washes in 0.1% TBS with 0.1% Tween, the membrane was incubated (1h, RT) with the proper secondary antibody HRP conjugated: goat anti-rabbit IgG (1:2000, Dako, Santa Clara, CA, USA) or goat anti-mouse IgG HRP conjugated (1:1500, Dako, Santa Clara, CA, USA).

Blots were revealed with Substrate Immobilion Western for HRP detection (Sigma-Aldrich, Saint-Quentin-Fallavier Cedex, France) and an ImageQuant Las 4000 mini (GE Healthcare, Chicago, IL, USA).
